# A Cross-Sectional Study of Antimicrobial Usage on Commercial Broiler and Layer Chicken Farms in Bangladesh

**DOI:** 10.3389/fvets.2020.576113

**Published:** 2020-12-16

**Authors:** Tasneem Imam, Justine S. Gibson, Mohammad Foysal, Shetu Bhusan Das, Suman Das Gupta, Guillaume Fournié, Md. Ahasanul Hoque, Joerg Henning

**Affiliations:** ^1^School of Veterinary Science, The University of Queensland, Gatton, QLD, Australia; ^2^Chattogram Veterinary and Animal Sciences University, Chattogram, Bangladesh; ^3^Department of Pathobiology and Population Sciences, Royal Veterinary College, University of London, London, United Kingdom

**Keywords:** antimicrobial usage, commercial chicken farms, broiler, layer, Chattogram, Bangladesh

## Abstract

Commercial poultry production is growing rapidly in Bangladesh to address the increasing demand for poultry meat and eggs. Challenges faced by producers include the occurrence of poultry diseases, which are usually treated or controlled by antimicrobials. A cross-sectional study was conducted on 57 commercial layer and 83 broiler farms in eight subdistricts of the Chattogram district, Bangladesh, to assess antimicrobial usage in relation to clinical signs observed in chicken flocks on these farms. Of the 140 commercial chicken farms, 137 (97.9%) used antimicrobials and 24 different antimicrobial agents were administered. On layer farms, the most commonly used antimicrobials were ciprofloxacin (37.0% of farms, 20/54), amoxicillin (33.3%, 18/54), and tiamulin (31.5%, 17/54), while on broiler farms, colistin (56.6%, 47/83), doxycycline (50.6%, 42/83), and neomycin (38.6%, 32/83) were most commonly administered. Only 15.3% (21/137) of farmers used antimicrobials exclusively for therapeutic purposes, while 84.7% (116/137) of farmers used them prophylactically, administering them either for prophylactic purposes only (22.6% of farmers, 31/137) or in combination with therapeutic purposes (62.1% of farmers, 85/137). About 83.3% (45/54) of layer farmers were selling eggs while antimicrobials were being administered compared to 36.1% (30/83) of the broiler farmers selling broiler chickens while administering antimicrobials. Overall, 75.2% (103/137) of farmers reported clinical signs for which they administered antimicrobials, while 24.8% (34/137) of farmers reported no clinical signs but still administered antimicrobials. Respiratory signs (71.8% of farms with clinical signs, 74/103) were most commonly reported, followed by enteric signs (32.0%, 33/103) and increased mortality (16.5%, 17/103). About 37.2% (51/137) of farmers bought antimicrobials exclusively from feed and chick traders, followed by veterinary medical stores (35.0%, 48/137). Purchasing antimicrobials from feed and chick traders was more common among broiler than layer farmers. It is recommended that commercial poultry farmers should keep records of antimicrobials used with dosage and duration of administration along with indication of use. This would allow farmers and veterinarians to review if antimicrobial usage had the desired effects and to evaluate the appropriate use of antimicrobial agents under an antimicrobial stewardship approach.

## Introduction

Poultry meat production has increased substantially over the past decades in South and South East Asia (1). In Bangladesh, where 20% of all protein consumed is derived from poultry products, most poultry species raised in Bangladesh are chickens (90%), followed by ducks (8%) and other species such as quail, pigeons, and geese (2%) (2).

Two poultry production systems are found in Bangladesh, commercial and backyard production. About 89% of households rear poultry with an average flock size of seven birds (2–4). Commercial chicken production can be classified into broiler and layer farming. In broiler farming, chickens are reared for meat while on layer farms, and chickens are reared for egg production although unproductive layer birds are also sold for meat (5).

The biggest challenge for commercial chicken producers is the occurrence of diseases (6). In Bangladesh, salmonellosis, colibacillosis, mycoplasmosis, and necrotic enteritis were the most frequent bacterial diseases reported from commercial chicken farms between 2002 and 2018, while infectious bursal disease, Newcastle disease, avian influenza, infectious bronchitis, avian leucosis, and fowl pox were the most common viral diseases reported during that period (7–11). Avian influenza in particular had a devastating effect on commercial chicken production in Bangladesh, resulting in a decrease of commercial chicken farms from 115,000 in 2007 to 55,000 in 2013 (12). Coccidiosis and ascaridiosis were the most common parasitic diseases reported in commercial poultry (7–11).

Thus, commercial poultry production requires comprehensive animal husbandry practises, which include antimicrobial therapy and vaccinations (13). Therapeutic application focuses on the treatment of birds with clinical signs of an infectious disease while prophylactic or preventive application refers to reduction of the risk of disease occurrence (14). In Bangladesh, antimicrobials are generally used for the treatment and prevention of poultry diseases, but some farmers use them also for growth promotion in order to increase feed conversion (15).

While the application of antimicrobials has contributed to the decline of mortality and morbidity rates in animals, misuse of antimicrobials is considered to be one of the biggest threats to human health (16). Antimicrobial resistance associated with inappropriate application of antimicrobials (17–20) can result in treatment failures for animal (21) and human diseases (22). This has significant economic and public health consequences, such as prolonged treatment duration of patients and longer hospital stays, which may further promote transmission of resistant pathogens in hospital (23), and represents an economic burden to the families of patients (24). The consumption of contaminated animal-source food (25, 26), direct contact with animals (27), or environmental exposure (28, 29) may promote the transmission of antimicrobial resistant bacteria to humans. In addition, animal-source food might contain antimicrobial residues if farmers do not adhere to recommended withholding periods for antimicrobial usage (30). Inappropriate use of antimicrobials in commercial chicken production is, therefore, a primary concern (17–20).

In Bangladesh, the extent of antimicrobial usage in livestock production is unknown (31) and data on national sales of antimicrobials are unreliable (32). In addition, frequent sales of antimicrobials through feed and chick traders and pharmaceutical company representatives (33) highlight the lack of governance on antimicrobial use in Bangladesh.

In the National Drug Policy 2016, the Bangladesh government published a list of priority drugs for the treatment of humans, which should not be sold “over the counter” (34). Unfortunately, a similar list of veterinary drugs not to be sold “over the counter” has not yet been published (34). Furthermore, there are neither regulations on veterinary drug registration and labelling (34) nor specific guidelines for the usage of antimicrobials in food animals available in Bangladesh (32). In addition, it has been shown that farmers in Bangladesh often do not follow the manufacturers' recommended dose and duration when administering antimicrobials to livestock (31).

Thus, this study aimed to assess (1) the frequency, purpose of usage, and sources of antimicrobials on commercial broiler and layer chicken farms in Chattogram, Bangladesh; (2) whether antimicrobial usage was associated with farmers' and farms' characteristics; (3) and the clinical signs for which antimicrobials were administered. In addition, compliance with the antimicrobials' withholding periods was evaluated.

## Materials and Methods

### Study Location

A cross-sectional study was conducted to collect data on antimicrobial usage on commercial broiler and layer farms in Chattogram district of Bangladesh.

The Chattogram district in the southeastern part of Bangladesh was selected as the study location because it is one of the main districts in the country in terms of chicken production. It is also the main region supplying chickens to Chattogram city, the second urban centre of the country (35). In 2014, 1,796 farmers in Chattogram reared commercial chickens on 289 layer and 1,507 broiler farms (36).

### Sampling Approach

**Figure 1:**
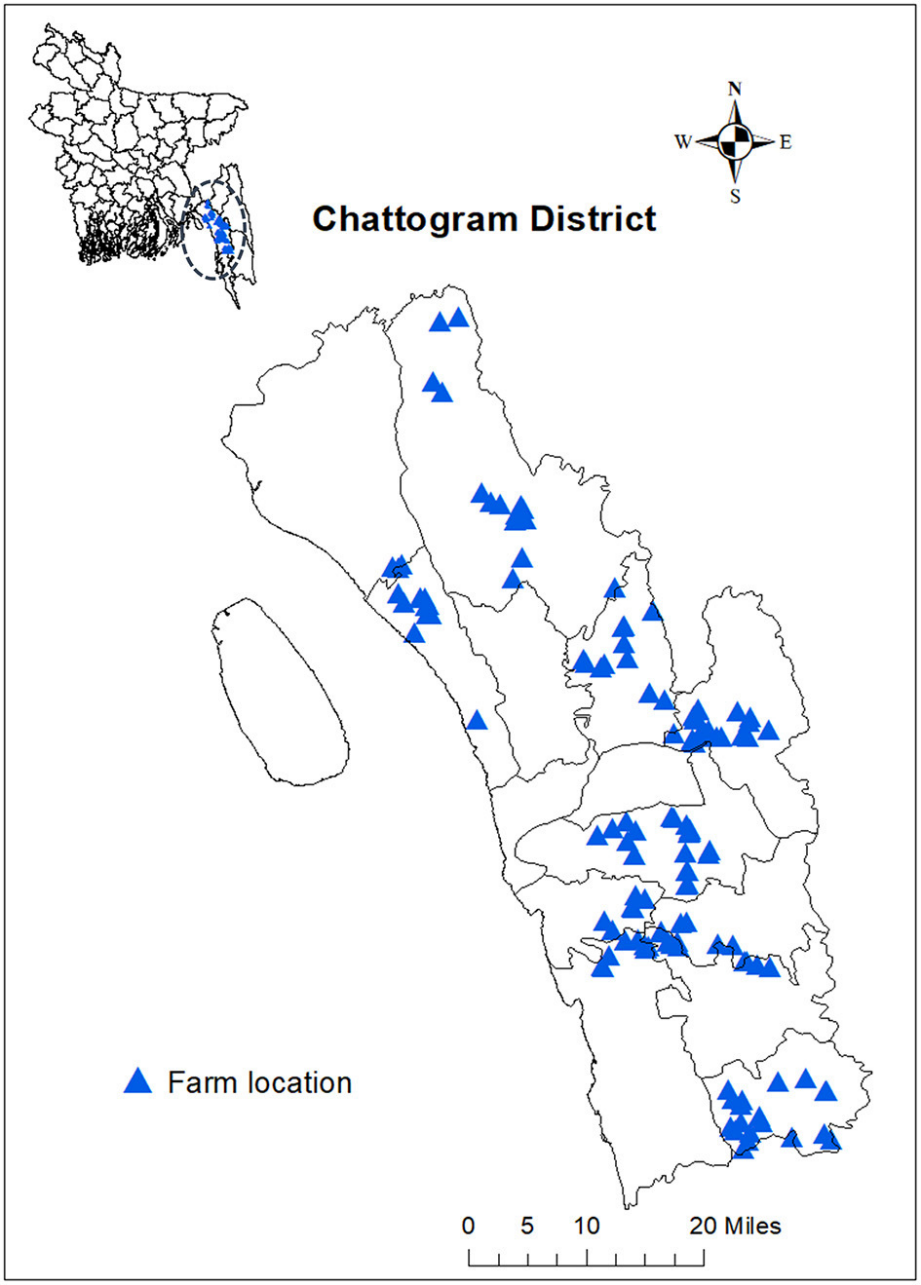
Location of studied commercial chicken farms in Chattogram, Bangladesh.

In the absence of a registry of commercial farms in the district, the farms included in this study were selected from a sampling frame of 1,748 commercial chicken farms that was created in 2017 (37). The sampling frame was developed by Gupta et al. (37) through consultation with the Bangladesh District Livestock Services, feed and chick traders, pharmaceutical representatives, and government and private practitioners. Gupta et al. (37) then selected farms using simple random sampling. The same farms were recruited in the current study, but some farms were excluded (*N* = 25) as they were no longer operating or had no chickens at the time of the field visits. Thus, 140 commercial chicken farms (83 broiler and 57 layer farms) in eight subdistricts (*upazilas*) of the Chattogram district were visited between February and May 2019 (Figure 1).

### Questionnaire and Interviews

A structured questionnaire was developed. It included sections on demographic/socioeconomic characteristics of the commercial chicken farmers and chicken management characteristics (flock size, chicken strains, number of sheds, age of chickens, type of the production system). It was piloted on five layer and five broiler farms.

Antimicrobial usage data was collected using a count-based approach, representing the use (yes/no) of an antimicrobial during the current production cycle (38). In addition, data on trade names of antimicrobials with active ingredients derived from trade name labels, purpose of antimicrobial application, frequency, dosage, duration, and route of administration of antimicrobials, adherence to withholding periods, sources from where antimicrobials were obtained, and sources of advice on antimicrobial administration and on sales of antimicrobials by chicken farmers were collected.

We also collected data on clinical signs and diseases observed by farmers in the current production cycle and information on biosecurity practises on farms and on attitudes and behaviours of farmers towards the usage of antimicrobials (Supplementary Material).

All collected data related to the current production period for one flock (poultry shed) at the time of the field visit.

When there were several chicken flocks or sheds, the following criteria were used to select the flock or the shed from which the data were collected:

- If the same number of antimicrobials was used in all sheds, the shed with the oldest chickens was selected.
- If the number of antimicrobials used across sheds differed, then the shed with the highest number of antimicrobials used was selected.

People most actively involved in chicken management on the visited farm were interviewed. Consent was obtained from the interviewees before the start of the interview. The interviews were conducted by a team of trained and experienced researchers from Chattogram Veterinary and Animal Sciences University (CVASU). All interviewers were fluent in English and Bangla. The interview was conducted in Bangla and responses were entered by the interviewers in the questionnaire in English. An interview lasted for about 1 h.

Although data on antimicrobial products used was obtained from the interviewees, photographs of antimicrobial packages were also taken to cross-check the information. Photographs of the drug registration book kept on the farms, if available, were also taken.

### Data Analysis

Frequencies were computed for farmer demographics (education, experience in poultry production) and antimicrobial usage (percentage of farms using antimicrobials, route of administration, source of antimicrobials, purpose of usage, adherence to withholding periods, sale of antimicrobials, and the occurrence of clinical signs on farms).

An association between any two categorical variables was investigated using a Fisher's exact test (39).

Univariable logistic regression models (40) were developed to assess whether using a given antimicrobial was associated with farm (farm type, flock size) and farmer characteristics (education, experience in poultry farming) the observation of a set of clinical signs during the current production cycle, the source of antimicrobials (veterinary medical stores, feed and chick traders), the purpose of usage, and the sale of antimicrobials by the farmer. Variables associated with a *p*-value < 0.2 were then considered for the multivariable analysis. A backward stepwise elimination procedure at a 5% level of significance was used to produce the final multivariable logistic regression model. The distributions of the outcome variables and predictors are shown in Supplementary Tables 5–12. Once a model with significant predictors was found, the confounding effect was examined by adding omitted predictors again and evaluating a change of the odds ratios by more than 20% (41). Biological plausible interactions were also evaluated in the final models. The overall model fit was examined by the Hosmer–Lemeshow test. The predictive ability of the models was evaluated by computing the area under the curve (AUC) of receiver operating characteristic (ROC) curves.

STATA 16 was used for the analysis (1985–2019, StataCorp). Farm locations were plotted across Chattogram district using ArcMAp 10.8 (ArcGIS, 1995–2019 Esri Inc.).

## Results

From 140 farms surveyed, 137 (97.9%) used antimicrobials. Data from these 137 farms, which comprised of 60.6% (83/137) broiler and 39.4% (54/137) layer farms, were analysed.

### Overview of the Study Population

#### Flock Characteristics

A total of 81.5% (44/54) of layer and 96.4% (80/83) of broiler farms had one shed only.

Layer flocks, with a median size of 2,150 chickens, were larger than broiler flock, with a median of 1,300 chickens (*p* < 0.001) (Table 1). The median age was 357 days for layer and 18 days for broiler flocks (Table 1).

**Table 1:** Socioeconomic and demographic characteristics of commercial layer and broiler chicken farmers and a description of the chicken farms in Chattogram, Bangladesh.

**Characteristics of the study population**			**Layer farmers (*N* = 54)**	**Broiler farmers (*N* = 83)**	**Fisher's exact *p*-value**
			**% (*N*)**	**% (*N*)**	
Characteristics of chicken farmers	Education level	No education/primary	7.4 (4)	21.7 (18)	0.032
		Secondary/graduate/post graduate	92.6 (50)	78.3 (65)	
	Experience in poultry raising	≤ 1 year	3.7 (2)	8.4 (7)	0.074
		1–5 years	11.1 (6)	26.5 (22)	
		6-10 years	24.1 (13)	15.7 (13)	
		>10 year	61.1 (33)	49.4 (41)	
	Poultry farming as the only source of income	No	31.5 (17)	41.0 (34)	0.283
		Yes	68.5 (37)	59.0 (49)	
	Sex	Male	100.0 (54)	98.8 (82)	1.000
		Female	0.0 (0)	1.2 (1)	
	Religion	Muslim	92.8 (77)	79.6 (43)	0.038
		Hindu	7.2 (6)	18.5 (10)	
		Buddhist	0.0 (0)	1.9 (1)	
Characteristics of chicken flocks	No. of sheds	1	81.5 (44)	96.4 (80)	0.006
		>1	18.5 (10)	3 (3.6)	
	Flock size	≤ 500	3.7 (2)	3.6 (3)	<0.001
		501–2500	55.6 (30)	86.6 (72)	
		>2500	40.7 (22)	9.6 (8)	
		Median flock size	2150 birds (Range: 120 to 7880)	1300 birds (Range: 120 to 4880)	
	Age	Mean age	334 days	19 days	
		Median age	357 days (Range: 26 to 720)	18 days (Range: 5 to 45)	

The most common layer breed was White Hyline Brown (61.1%, 33/54), followed by Novogen Brown (16.7%, 9/54), Bovans Brown (7.4%, 4/54), White Shaver 579 (5.6%, 3/54), and Hisex Brown (3.7%, 2/54). On broiler farms, Indian River (39.8%, 33/83) and Cobb 500 (38.6%, 32/83) were the most common breeds, followed by 12.0% of Hubbard Classic (10/83) and 8.4% of Ross 308 (7/83). However, for 5.6% (3/54) of the layer and 1.2% (1/83) of the broiler farms the breed was not specified.

#### Farmer Characteristics

People most actively involved in chicken management on the visited farm were interviewed. In most cases, they were either the farm owner (77.4%, 106/137), or the main farm worker (22.6%, 31/137).

More layer farmers (92.6%, 50/54) had a higher educational qualification (secondary, higher secondary, graduate, or postgraduate level) than broiler farmers (78.3%, 65/83) (*p* = 0.032) (Table 1).

Layer farmers were marginally more experienced in poultry farming compared to broiler farmers (*p* = 0.074) (Table 1).

### Antimicrobial Usage

#### Antimicrobial Agents, Their Purpose, and Route of Administration

A total of 24 different antimicrobial agents were administered in the current production cycle at the time of the study in 449 different ways (either alone or in combination with other antimicrobials) (Supplementary Table 1). Eight of these 24 antimicrobials were most commonly applied (either alone or in combination with other antimicrobials), representing 71.5% (321/449) of the overall usage of antimicrobials on the farms. These eight antimicrobials (colistin, ciprofloxacin, tylosin, neomycin, amoxicillin, trimethoprim sulfonamides, doxycycline, and tiamulin) represent the most frequently used antimicrobials in each of the eight antimicrobial classes (polymyxins, quinolones, macrolides, aminoglycosides, beta lactams, tetracyclines, sulfonamides, and pleuromutilins). Further data analysis focused on these eight antimicrobials.

On layer farms, the most commonly administered antimicrobial was ciprofloxacin 37.0% (20/54), followed by amoxicillin 33.3% (18/54) and tiamulin 31.5% (17/54). On broiler farms, the most commonly administered antimicrobial was colistin 56.6% (47/83), followed by doxycycline 50.6% (42/83) and neomycin 38.6% (32/83). Doxycycline, neomycin and colistin were more frequently applied on broiler farms compared to layer farms (*p* < 0.05), while tiamulin was not used on broiler farms (Table 2).

**Table 2:** Antimicrobials used (according to medical importance) on commercial layer and broiler farms in Chattogram, Bangladesh.

**Importance of antimicrobials^**a**^**	**Usage of antimicrobials on commercial chicken farms**^**b**^		**Layer farmers (*N* = 54)**	**Broiler farmers (*N* = 83)**	**Fisher's exact test *p*-value**
			**% (*N*)**	**% (*N*)**	
Highest Priority Critically Important	Colistin	Yes	27.8 (15)	56.6 (47)	0.001
		No	72.2 (39)	43.4 (36)	
	Ciprofloxacin	Yes	37.0 (20)	33.7 (28)	0.717
		No	63.0 (34)	66.3 (55)	
	Tylosin	Yes	16.7 (9)	20.5 (17)	0.659
		No	83.3 (45)	79.5 (66)	
High-Priority Critically Important	Neomycin	Yes	7.4 (4)	38.6 (32)	<0.001
		No	92.6 (50)	61.4 (51)	
	Amoxicillin	Yes	33.3 (18)	32.5 (27)	1.000
		No	66.7 (36)	67.5 (56)	
Highly Important	Trimethoprim sulfonamides (SXT)	Yes	29.6 (16)	18.1 (15)	0.144
		No	70.4(38)	81.9 (68)	
	Doxycycline	Yes	25.9 (14)	50.6 (42)	0.005
		No	74.1 (40)	49.4 (41)	
Important	Tiamulin^c^	Yes	31.5 (17)	0.0 (0)	<0.001
		No	68.5 (37)	100.0 (83)	

*Usage relates to the application of antimicrobials in the current production cycle at the time of the survey in 2019 (mean age of layers: 334 days, mean age of broilers: 19 days)*.

a *Classified as per WHO Critically Important Antimicrobials for Human Medicine 6th revision*.

b *Most frequently used antimicrobials, representing 71.5% of overall usage*.

c *Tiamulin was not used on commercial broiler farms during the time of the survey*.

**Figure 2:**
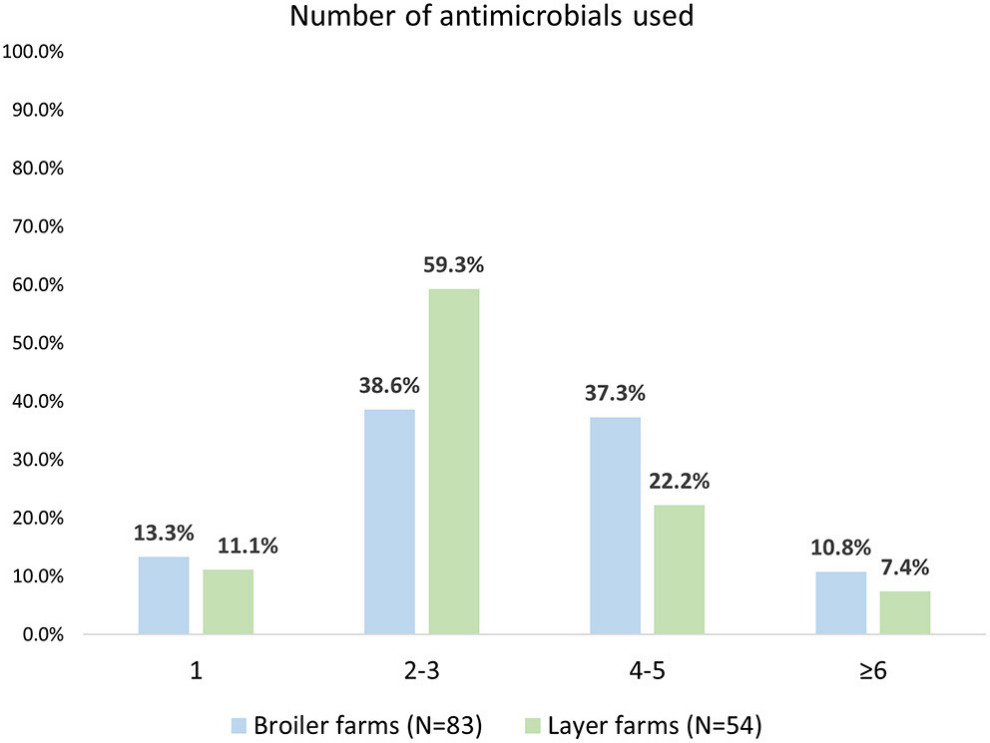
Number of antimicrobials used on layer and broiler farms in Chattogram, Bangladesh.

The number of antimicrobials used on farms was categorised in the following groups: 1, 2–3, 4–5, 6, or more. Overall, these (categorised) number of antimicrobials used did not differ between layer and boiler farms (*p* = 0.120). However, most farmers used 2–3 antimicrobials (59.3% of layer and 38.6% of broiler farmers), while usage of 4–5 antimicrobials was more common on broiler than on layer farms (37.3% vs. 22.2%) (Figure 2).

Antimicrobials were administered in water by 97.1% (133/137) of farmers (with 91.3% (125/137) of farmers providing them in water only and 5.8% (8/137) in both water and feed) and in feed by 8.7% (12/137) of farmers (with 2.9% (4/137) of farmers providing it in feed only).

Only 15.3% (21/137) of farmers used antimicrobials exclusively for therapeutic purposes, while 84.7% (116/137) of farmers used them prophylactically, administering them either for prophylactic purposes only (22.6% (31/137) of farmers) or in combination with therapeutic purposes (62.1% (85/137) of farmers). The purpose of using antimicrobials did not differ between layer and broiler farms (*p* = 0.328, Supplementary Table 2). None of the farmers indicted they used antimicrobials as growth promoters.

Eggs and birds were sold while antimicrobials were still administered in flocks, with 83.3% (45/54) of layer farmers selling eggs, and 36.1% (30/83) of broiler farmers selling birds while administering antimicrobials (*p* < 0.001). The most common antimicrobials used on layer farms while selling eggs were ciprofloxacin (40.0%, 18/45), trimethoprim sulfonamides (35.6%, 16/45), and amoxicillin (33.3%, 15/45). On broiler farms, colistin (66.7%, 20/30), doxycycline (53.3%, 16/30), and ciprofloxacin (46.7%, 14/30) were the most common antimicrobials administered, while broilers were sold. Except for trimethoprim sulfonamide usage on layer farms, there was no difference at *p* < 0.05 between farms selling eggs or broilers and not selling eggs or broilers while administering antimicrobials for the type of antimicrobials used (Supplementary Table 3). Broiler farmers (63.9%, 53/83) who stopped using antimicrobials before selling their birds stopped on average 4.2 (95% CI: 3.6, 4.8) days (Median: 3) before the sale of birds.

#### Clinical Signs for Which Antimicrobials Were Used

Overall, 75.2% (103/137) of farmers reported clinical signs (alone or in combination) during the production period, while 24.8% (34/137) of farmers did not observe any clinical signs of disease.

Antimicrobials were most frequently used for respiratory signs (alone or in combination with other signs) (71.8%, 74/103), followed by enteric signs (32.0%, 33/103). Antimicrobials were used to address increased mortality (alone or in combination with other signs) on 16.5% (17/103) of farms, while 16.5% (17/103) of farmers administered antimicrobials to prevent and/or treat swollen head, ascites, in-appetence, and eye problems. Decreased egg production or poor-quality eggs were specified by 20.4% (11/54) of layer farmers as a reason for using antimicrobials (Supplementary Table 4).

**Figure 3:**
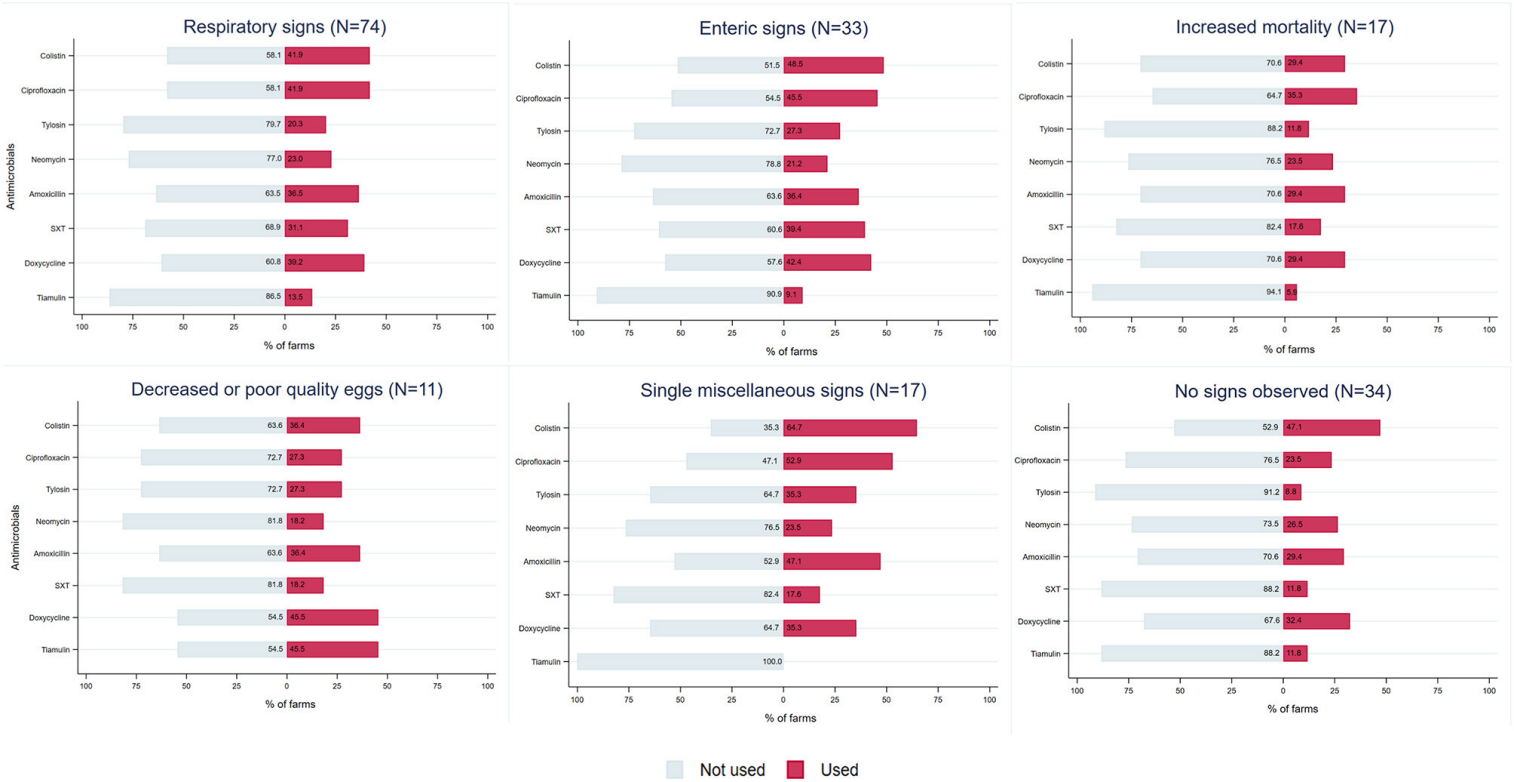
Types of antimicrobial used (percent usage) by commercial layer and broiler farmers in Chattogram, Bangladesh, for clinical signs reported on these farms. Usage relates to the application of antimicrobials in the current production cycle at the time of the survey in 2019 (mean age of layers: 334 days, mean age of broilers: 19 days). Most frequently used antimicrobials are presented here, representing 71.5% of overall usage.

Colistin and ciprofloxacin were the most frequently used antimicrobials on farms reporting respiratory signs (41.9%, 31/74; 41.9%, 31/74), enteric signs (48.5%, 16/33; 45.5%, 15/33), and increased mortality (29.4%, 5/17; 35.3%, 6/17), as well as single miscellaneous signs, such as swollen head, ascites, in-appetence, and/or eye problem. However, doxycycline (45.5%, 5/11) and tiamulin (45.5%, 5/11) were preferred to address a reduction in egg production. In the absence of clinical signs, colistin (47.1%, 16/34), doxycycline (32.4%, 11/34), and amoxicillin (29.4%, 10/34) were the most frequent antimicrobials administered (Figure 3).

#### Source of Antimicrobials

Farmers bought antimicrobials most frequently (sole source or in combination with other sources) from feed and chick traders (43.8%, 60/137), veterinary medical stores (44.5%, 61/137), and pharmaceutical representatives (5.1%, 7/137). More specifically, farmers bought antimicrobials only from feed and chick traders in 37.2% (51/137) of cases and from veterinary medical stores only in 35.0% of cases (48/137). Broiler farmers were more likely to purchase antimicrobials only from feed and chick traders (45.8%, 38/83) compared to layer farmers (24.1%, 13/54) (*p* = 0.012).

The usage of individual antimicrobials on farms was not associated with the supply of antimicrobials through feed and chick traders (*p* > 0.05) Supplementary Tables 5-12. Doxycycline and colistin were the antimicrobials used by farmers that were less likely purchased from veterinary medical stores (*p* = 0.017 and *p* = 0.041, respectively) Supplementary Tables 6, 11.

About 16.1% (22/137) of farmers sold antimicrobials to other farmers.

#### Factors Associated With Antimicrobial Usage

Univariable logistic regression models for each of the most commonly used antimicrobials, namely, colistin, ciprofloxacin, tylosin, neomycin, amoxicillin, trimethoprim sulfonamides, doxycycline, and tiamulin, are shown in Supplementary Tables 5-12.

Factors with *p*-values < 0.05 in the multivariable models were identified for trimethoprim sulfonamides and neomycin.

Farmers were 3.1 times (95% CI: 1.3–7.8) more likely to administer trimethoprim sulfonamides for respiratory signs (Table 3). Farmers were also 3.1 (95% CI: 1.3–7.7) more likely to use trimethoprim sulfonamides for enteric signs (Table 3). The model for trimethoprim sulfonamides usage showed a good fit (Hosmer–Lemeshow *p*-value = 0.681, AUC = 0.68).

**Table 3:** Results of the multivariable analysis for risk factors associated with the use of trimethoprim sulfonamides on commercial chicken farms in Bangladesh in 2019.

**Risk factors**	**Category**	**OR^**a**^ (95% CI^**b**^)**	**Logistic regression *p*-value**
Occurrence of respiratory signs	No	Ref	0.014
	Yes	3.1 (1.3–7.8)	
Occurrence of enteric signs	No	Ref	0.012
	Yes	3.1 (1.3–7.7)	

a *Odds ratio*.

b *Confidence interval*.

The odds of using neomycin was higher in broiler than layer farms (OR = 8.3, 95% CI = 2.7–25.6) and among farmers selling antimicrobials (OR = 3.2, 95% CI = 1.1–9.1) (Table 4). The model for neomycin usage showed a good fit (Hosmer–Lemeshow p-value = 0.201, AUC = 0.74).

**Table 4:** Results of the multivariable analysis for risk factors associated with the use of neomycin on commercial chicken farms in Bangladesh in 2019.

**Risk factors**	**Category**	**OR^**a**^ (95% CI^**b**^)**	**Logistic regression *p*-value**
Farm type	Layer	Ref	<0.001
	Broiler	8.3 (2.7–25.6)	
Sale of antimicrobials to other commercial chicken farmers	No	Ref	0.030
	Yes	3.2 (1.1–9.1)	

a *Odds ratio*.

b *Confidence interval*.

## Discussion

Our study highlighted that usage of antimicrobials is very common in the commercial poultry industry of Bangladesh, with almost all broiler and layer farmers administrating antimicrobials to their flocks. Use of medically important antimicrobials, non-adherence to withholding periods, usage of antimicrobials despite the non-occurrence of any clinical signs, and sales of antimicrobials without veterinary advice were frequently reported.

There is little reliable data on the extent of antimicrobials usage in the livestock production system across South East Asia (42–44) and in particular, from commercial poultry farms in Bangladesh (15, 32). We were able to provide detailed data of antimicrobial usage in the commercial layer and broiler industry of Bangladesh. In addition, while previous studies used convenience sampling to describe antimicrobial usage on commercial poultry farms (45, 46), we used a random sampling approach, increasing the external validity of our study findings.

The majority of farmers used antimicrobials for prophylactic purposes. Prophylactic administration of antimicrobials may be conducted to compensate for substandard farm management conditions, to prevent frequently occurring poultry diseases (13) or because vaccinations against poultry diseases were not conducted. Furthermore, cost associated with veterinary treatments (47) might result in farmers administering drugs prophylactically in order to prevent severe clinical events that require substantial, and expensive, veterinary interventions. Farmers may also have difficulties in accessing veterinary services to diagnose diseases. Laboratory confirmation of livestock, including poultry, diseases in Bangladesh is only conducted in District Veterinary Hospitals, Regional Field Diseases Investigation Laboratories, and the Central Disease Investigation Laboratory of Department of Livestock Services (48), which represents only a small number of laboratories compared to the number of poultry farms in Bangladesh. Thus, farmers might be unable to use these laboratories to diagnose diseases from samples collected, which could result in a widespread prophylactic administration of antimicrobials based on farmers' perceptions of disease risk or their own experience.

It has been shown that antimicrobial usage on commercial poultry farms is strongly driven by advice provided from antimicrobial suppliers; in particular, feed and chick traders who closely work with representatives of drug companies to achieve target sales, may have influenced farmers' behaviours in using antimicrobials (33). A large proportion of commercial chicken farmers were supplied with antimicrobials from feed and chick traders. The use of antimicrobials may be influenced by contractual agreements with the feed and chick traders who supply all production inputs (e.g., day-old chicks and feed) in credit to farmers and then purchase the poultry products from farmers at pre-arranged prices (33). This practise is more common among broiler than layer farmers; thus, these transactional arrangements likely explain the differences observed between the two production types in this study.

Farmers did not report the use of antimicrobials as growth promoters. It has been suggested that the use of antimicrobials for growth promotion might represent a considerable cost for farmers, and therefore, they might refrain from using antimicrobials for this purpose (15).

Eight antimicrobials were most frequently administered in our study area (either alone or in combination with other antimicrobials). These antimicrobials are commonly used in the poultry industry and included colistin, ciprofloxacin, and tylosin, which are considered as “Highest Priority Critically Important Antimicrobials” for public health (49). Indeed, Colistin is used as last resort for the treatment of infections with *Enterobacteriaceae* and *Pseudomonas aeruginosa*, while tylosin is used for *Legionella, Campylobacter* spp., MDR *Salmonella* spp., and *Shigella* infections, and ciprofloxacin for the treatment of *Campylobacter* spp. and *Salmonella* spp. infections (50). It is recommended that ciprofloxacin and colistin should not to be administered in animals as first-line therapy and should only be used after obtaining culture and susceptibility test results. In fact, ciprofloxacin and colistin should not be administered at all to any food-producing animals including chickens in the absence of any clinical signs (51). Surprisingly, many farmers used antimicrobials (including ciprofloxacin, tylosin, and colistin) without observing any clinical signs, and even when they did observe some, the decision to use those antimicrobials was rarely informed by veterinarians.

The use of colistin, doxycycline, and neomycin was higher on broiler farms compared to layer farms. Doxycycline was also more commonly administered by less experienced broiler farmers and was often administered in combination with other antimicrobials, including colistin. The frequent use of colistin may reflect the fact that they are considered as an “essential” antimicrobial in the poultry industry and have been used there for a long time (52).

Neomycin was more frequently used on broiler farms and by farmers who sell antimicrobials. The relatively low cost of neomycin may be the reason for its frequent usage (53) but also its re-sale by commercial farmers.

Similar to previous research, farmers in our study mostly mixed antimicrobials into water (33). This is in accordance with the Bangladesh Fish Feed and Animal Feed Act 2010, which highlights that antimicrobials are not permitted to be added into feed (54). However, some farmers in our study were breaching the Act by administering antimicrobials in feed.

Layer farmers reported selling eggs and broiler farmers selling chickens while administering antimicrobials. Antimicrobial residues was previously found in poultry in Bangladesh (55–57). For instance, trimethoprim sulfonamides, which were frequently used by layer farmers while selling eggs, are not approved to be used in laying chickens as the trimethoprim residues can be detected in egg yolk as well as albumen for more than 7 days after its administration (58). Farmers in Bangladesh may not be aware of withholding periods and the residual effects of antimicrobials (32). There may also be a lack of information from veterinarians on withdrawal periods of antimicrobials (32). Furthermore, continuous occurrence of clinical signs might result in farmers deciding to constantly use antimicrobials on their chickens until the time of sale of their poultry products (59).

A lack of monitoring from governmental agencies had been previously identified as a reason why withholding periods were not adhered to by farmers (32). This includes monitoring of farm management practises but also monitoring antimicrobial residues according to Codex standards (60). Unfortunately, there are limited facilities in Bangladesh to conduct residue analysis in tissues of animal origin (61). Establishing government or private laboratories with infrastructure and expertise in identifying residues will assist in residue detection and in monitoring appropriate antimicrobial use. However, it is uncertain if farmers would actually submit samples for residue testing—such residue monitoring is usually conducted by regulatory bodies.

In Bangladesh, only registered veterinarians are authorised to prescribe antimicrobials as per the Bangladesh Veterinary Practitioners Ordinance, 1982 (62) and only registered pharmacists are permitted to sell antimicrobials with a prescription as per Drug Act 1940 (63). Thus, veterinarians only prescribe, but do not sell, antimicrobials in Bangladesh. However, in practise, antimicrobials are available in Bangladesh “over the counter” from veterinary medical stores without prescriptions.

Sixteen percent of farmers sold antimicrobials to other farmers. These arrangements make antimicrobials very accessible in Bangladesh and increase the risk of their improper use on poultry farms. Easy access to antimicrobials is not unique to Bangladesh and has also been described for other Southeast Asian countries such as India, Indonesia, Nepal, Bhutan, Thailand, Sri Lanka, and Maldives (44).

Our study had a number of limitations. Firstly, due to the retrospective nature of the data collection, we could not observe the clinical signs and relied on farmers' reports. Recall bias might have also impacted the reporting by farmers. Furthermore, this study only collected data on clinical signs reported during the production period and antimicrobials used in that production period. However, farmers did not keep records of the antimicrobials they used in response to which clinical signs and for what duration. We collected data on the dosage of antimicrobials administered to chickens, but the data quality was poor and did not permit a reliable analysis. A prospective study with detailed (daily) observations on clinical signs, diseases antimicrobial usage (including dosage and duration), and treatment outcomes would be able to better explore the association between antimicrobial usage and the motivations for its application.

Based on the results of this study, it is recommended that farmers should keep records of antimicrobials used with dosage and duration of administration along with the use of specific antimicrobials against which diseases or clinical signs. This would certainly allow farmers and veterinarians to evaluate if antimicrobial usage had the desired outcome and allow adherence to withholding periods.

Education or extension programs for poultry farmers on the use of antimicrobials are highly warranted. Such training should encompass information on withholding periods for antimicrobial usage and should highlight the importance of vaccinations to control viral and bacterial infections in poultry. The association between good biosecurity and infection control practises and diseases needs to be highlighted in order to reduce the further use of antimicrobials. Extension programs are implemented in Bangladesh by government and nongovernmental agencies. The Department of Livestock and Services in Bangladesh drafted a “National Livestock Extension Policy 2013” and highlighted the importance of establishing collaborative livestock extension services that include all stakeholders (64). In case of antimicrobial applications in the poultry industry, this would include, in addition to the poultry farmers, also suppliers of antimicrobials (e.g., feed and chick traders and veterinary medical representatives).

Changing regulatory frameworks is most challenging. Currently, no enforced national strategy for the control of antimicrobials in food animals exists for Bangladesh. The Ministry of Health and Family Welfare has developed a national action plan (2017–2022) for antimicrobial resistance containment in Bangladesh (65), but unfortunately relevant policies have not been implemented yet (66).

Also, prohibiting over-the-counter sales of antimicrobials without the prescription from a registered veterinarian is not enforced in Bangladesh. Recently, a ruling of the High Court Division of the Supreme Court of Bangladesh highlighted that sales of antimicrobials should only be conducted with prescription (67). It is recommended to closely work with farmers' to evaluate societal factors influencing poultry management practises in order to develop evidence-based and practical policies for farmers to reduce and modify antimicrobial usage.

In conclusion, our research highlights the challenges faced by commercial poultry producers in Bangladesh and outlined opportunities to improve the appropriate use of antimicrobial agents under an antimicrobial stewardship approach.

## Data Availability Statement

The raw data supporting the conclusions of this article will be made available by the authors, without undue reservation.

## Ethics Statement

The studies involving human participants were reviewed and approved by Human Ethics. Approval for the interviews was obtained from the University of Queensland Institutional Human Ethics Committee on the 7 December 2018 (Approval number: 2018002266). Written informed consent for participation was not required for this study in accordance with the national legislation and the institutional requirements. Ethical review and approval was not required for the animal study because the respondents were farmers of commercial chicken farms.

## Author Contributions

JH, GF, and MH designed the research study and obtained the funding for the field research. The questionnaire was developed by TI with inputs from JH, JG, and SG. The data collection strategy was developed by JH, GF, MH, TI, and SG. Data collection was conducted by MF and SD. TI conducted data analysis under the guidance of JH, GF, and JG. TI prepared the initial draft, figures, tables, and supplementary materials, with edits provided by JH and JG. All authors have read, contributed to, and approved the final version of the manuscript.

## Conflict of Interest

The authors declare that the research was conducted in the absence of any commercial or financial relationships that could be construed as a potential conflict of interest.
