# Supplementary material for: A Cross-Sectional Study of Antimicrobial Usage on Commercial Broiler and Layer Chicken Farms in Bangladesh
Source: Front Vet Sci. 2020 Dec 16;7:576113. doi: 10.3389/fvets.2020.576113 (PMC7772320; doi:10.3389/fvets.2020.576113)
Supplement: Supplementary file 1 [file Data_Sheet_1.docx]

**Supplementary Materials**

**Table S1**. Antimicrobials used on commercial layer and broiler farms in Chattogram, Bangladesh. Usage relates to the application of antimicrobials in the current production cycle at the time of the survey in 2019 (mean age of layers: 334 days, mean age of broilers: 19 days).

| **Antimicrobials used on commercial chicken farms** | | **Layer farms (N=54)** | **Broiler farms (N=83)** | **Fisher's exact test**  **p-value** |
| --- | --- | --- | --- | --- |
|  |  | **% (N)** | **% (N)** |  |
| Amoxicillin | Yes | 33.3 (18) | 35.2 (27) | 1.000 |
|  | No | 66.7 (36) | 67.5 (56) |  |
| Doxycycline | Yes | 25.9 (14) | 50.6 (42) | 0.005 |
|  | No | 74.1 (40) | 49.4 (41) |  |
| Oxytetracycline | Yes | 14.8 (8) | 16.9 (14) | 0.816 |
|  | No | 85.2 (46) | 83.1 (69) |  |
| Chlortetracycline | Yes | 14.8 (8) | 2.4 (2) | 0.014 |
|  | No | 85.2 (46) | 97.6 (81) |  |
| Ciprofloxacin | Yes | 37.0 (20) | 33.7 (28) | 0.717 |
|  | No | 63.0 (34) | 66.3 (55) |  |
| Enrofloxacin | Yes | 9.3 (5) | 19.3 (16) | 0.147 |
|  | No | 90.7 (49) | 80.7 (67) |  |
| Levofloxacin | Yes | 9.3 (5) | 4.8 (4) | 0.316 |
|  | No | 90.7 (49) | 95.2 (79) |  |
| Norfloxacin | Yes | 7.4 (4) | 2.4 (2) | 0.212 |
|  | No | 92.6 (50) | 97.6 (81) |  |
| Pefloxacin | Yes | 1.9 (1) | 1.2 (1) | 1.000 |
|  | No | 98.1 (53) | 98.8 (82) |  |
| Flumequine | Yes | 3.7 (2) | 6.0 (5) | 0.704 |
|  | No | 96.3 (52) | 94.0 (78) |  |
| Erythromycon | Yes | 9.3 (5) | 10.8 (9) | 1.000 |
|  | No | 90.7 (49) | 89.2 (74) |  |
| Azithromycin | Yes | 1.9 (1) | 1.2 (1) | 1.000 |
|  | No | 98.1 (53) | 98.8 (82) |  |
| Tylosin | Yes | 16.7 (9) | 20.5 (17) | 0.659 |
|  | No | 83.3 (45) | 79.5 (66) |  |
| Tilmicosin | Yes | 7.4 (4) | 4.8 (4) | 0.712 |
|  | No | 92.6 (50) | 95.2 (79) |  |
| Sulphur drugs | Yes | 5.6 (3) | 4.8 (4) | 1.000 |
|  | No | 94.4 (51) | 95.2 (79) |  |
| Trimethoprim sulphonamides | Yes | 29.6 (16) | 18.1 (15) | 0.144 |
|  | No | 70.4 (38) | 81.9 (68) |  |
| Gentamicin | Yes | 1.9 (1) | 9.6 (8) | 0.088 |
|  | No | 98.1 (53) | 90.4 (75) |  |
| Neomycin | Yes | 7.4 (4) | 38.6 (32) | <0.001 |
|  | No | 92.6 (50) | 61.4 (51) |  |
| Spiramycin | Yes | 0.0 (0) | 2.4 (2) | 0.519 |
|  | No | 100.0 (54) | 97.6 (81) |  |
| Colistin | Yes | 27.8 (15) | 56.6 (47) | 0.001 |
|  | No | 72.2 (39) | 43.4 (36) |  |
| Tiamulin | Yes | 31.5 (17) | 0.0 (0) | <0.001 |
|  | No | 68.5 (37) | 100.0 (83) |  |
| Lincomycin | Yes | 1.9 (1) | 2.4 (2) | 1.000 |
|  | No | 98.1 (53) | 97.6 (81) |  |
| Fosfomycin | Yes | 3.7 (2) | 0.0 (0) | 0.154 |
|  | No | 96.3 (52) | 100.0 (83) |  |
| Florfenicol | Yes | 0.0 (0) | 4.8 (4) | 0.153 |
|  | No | 100.0 (54) | 95.2 (79) |  |

**Table S2** Purpose of antimicrobial usage on commercial layer and broiler farms in Chattogram, Bangladesh. No farmers indicated to use antimicrobials as a growth promoter.

| **Purpose of using antimicrobials** | **Layer farms**  **(N=54)** | **Broiler farms (N=83)** | **Fisher's exact test**  **p-value** |
| --- | --- | --- | --- |
|  | **% (N)** | **% (N)** |  |
| Therapeutic | 20.4 (11) | 12.0 (10) | 0.328 |
| Prophylactic | 24.1 (13) | 21.7 (18) |  |
| Both therapeutic and prophylactic | 55.6 (30) | 66.3 (55) |  |

**Table S3.** Antimicrobials administered on commercial layer and broiler farms in Chattogram, Bangladesh, while eggs or broilers being sold from these farms.

| **Antimicrobials used on commercial chicken farms^a^** | | **Layer farmers selling eggs while using antimicrobials (N=54)** | | | **Broiler farmers selling broilers while using antimicrobials (N=83)** | | |
| --- | --- | --- | --- | --- | --- | --- | --- |
|  |  | **Yes**  **(N=45)** | **No**  **(N=9)** | **Fisher's**  **exact test**  **p-value** | **Yes**  **(N=30)** | **No**  **(N=53)** | **Fisher's exact test**  **p-value** |
|  |  | **% (N)** | **% (N)** |  | **% (N)** | **% (N)** |  |
| Colistin | Yes | 26.7 (12) | 33.3 (3) | 0.696 | 66.7 (20) | 50.9 (27) | 0.177 |
|  | No | 73.3 (33) | 66.7 (6) |  | 33.3 (10) | 49.1 (26) |  |
| Ciprofloxacin | Yes | 40.0 (18) | 22.2 (2) | 0.458 | 46.7 (14) | 26.4 (14) | 0.090 |
|  | No | 60.0 (27) | 77.8 (7) |  | 53.3 (16) | 73.6 (39) |  |
| Tylosin | Yes | 15.6 (7) | 22.2 (2) | 0.635 | 16.7 (5) | 22.6 (12) | 0.583 |
|  | No | 84.4 (38) | 77.8 (7) |  | 83.3 (25) | 77.4 (41) |  |
| Neomycin | Yes | 6.7 (3) | 11.1 (1) | 0.529 | 43.3 (13) | 35.8 (19) | 0.639 |
|  | No | 93.3 (42) | 88.9 (8) |  | 56.7 (17) | 64.2 (34) |  |
| Amoxicillin | Yes | 33.3 (15) | 33.3 (3) | 1.000 | 36.7 (11) | 30.2 (16) | 0.628 |
|  | No | 66.7 (30) | 66.7 (6) |  | 63.3 (19) | 69.8 (37) |  |
| Trimethoprim  sulphonamides | Yes | 35.6 (16) | 0.0 (0) | 0.045 | 16.7 (5) | 18.9 (10) | 1.000 |
|  | No | 64.4 (29) | 100.0 (9) |  | 83.3 (25) | 81.1 (43) |  |
| Doxycycline | Yes | 24.4 (11) | 33.3 (3) | 0.681 | 53.3 (16) | 49.1 (26) | 0.820 |
|  | No | 75.6 (34) | 66.7 (6) |  | 46.7 (14) | 50.9 (27) |  |
| Tiamulin^b^ | Yes | 33.3 (15) | 22.2 (2) | 0.703 | - | - | - |
|  | No | 66.7 (30) | 77.8 (7) |  | - | - |  |

^a^ Most frequently used antimicrobials, representing 71.5% of overall usage.

^b^ Tiamulin was not used on commercial broiler farms.

**Table S4.** Clinical signs observed by commercial layer and broiler farms in Chattogram, Bangladesh, for which antimicrobials were used (N=103. Usage relates to the application of antimicrobials in the current production cycle at the time of the survey in 2019 (mean age of layers: 334 days, mean age of broilers: 19 days).

| **Clinical signs observed on commercial chicken farms (N=103)^a^** | **% (N) of farmers using antimicrobials** |
| --- | --- |
| No clinical signs observed | 24.8 (34) |
| Respiratory signs | 71.8 (74) |
| Enteric signs | 32.0 (33) |
| Increased mortality | 16.5 (17) |
| Single miscellaneous signs^b^ | 16.5 (17) |
| **Clinical signs observed on layer farms (N=54)** |  |
| Decreased egg production or poor quality | 20.4 (11) |

^a^ Column % total is not 100% because farmers used antimicrobials for multiple reasons.

^b^ This includes swollen head, ascites, in-appetence, and eye problems

**Table S5**. Results of the univariate analysis for risk factors associated with the use of amoxicillin on commercial poultry farms in Bangladesh in 2019.

| **Risk factors** | **Category** | **Use of Amoxicillin** | | **OR**  **(95% CI)** | **Logistic regression p-value** |
| --- | --- | --- | --- | --- | --- |
|  |  | **Yes** | **No** |  |  |
|  |  | **% (N)** | **% (N)** |  |  |
| Farm type | Layer | 33.3 (18) | 66.7 (36) | Ref | 0.922 |
|  | Broiler | 32.5 (27) | 67.5 (56) | 0.9 (0.5-2.0) |  |
| Flock size | ≤500 | 20.0 (1) | 80.0 (4) | Ref | 0.752 |
|  | 501-2500 | 32.4 (33) | 67.7 (69) | 1.9 (0.2-17.8) |  |
|  | >2500 | 11.0 (11) | 63.3 (19) | 2.3 (0.2-23.4) |  |
| Education | No education/  primary | 35.7 (41) | 64.4 (74) | Ref | 0.119 |
|  | Secondary/graduate/  post graduate | 18.2 (4) | 81.8 (18) | 2.5 (0.8-7.9) |  |
| Experience | 6 months -5 years | 29.7 (11) | 70.3 (26) | Ref | 0.637 |
|  | >5 years | 34.0 (34) | 66.0 (66) | 1.2 (0.5-2.8) |  |
| Presence of any clinical signs reported | No | 29.4 (10) | 70.6 (24) | Ref | 0.623 |
|  | Yes | 34.0 (35) | 66.0 (68) | 0.8 (0.3-1.9) |  |
| Occurrence of respiratory signs | No | 28.6 (18) | 71.4 (45) | Ref | 0.326 |
|  | Yes | 36.5 (27) | 63.5 (47) | 1.4 (0.7-3.0) |  |
| Occurrence of enteric signs | No | 31.7 (33) | 68.3 (71) | Ref | 0.622 |
|  | Yes | 36.4 (12) | 63.6 (21) | 1.2 (0.5-2.8) |  |
| Increased mortality | No | 33.3 (40) | 66.7 (80) | Ref | 0.748 |
|  | Yes | 29.4 (5) | 70.6 (12) | 0.8 (0.3-2.5) |  |
| Decreased egg production or poor quality | No | 67.5 (85) | 32.5 (41) | Ref | 0.796 |
|  | Yes | 63.6 (7) | 36.4 (4) | 1.2 (0.3-4.3) |  |
| Occurrence of single miscellaneous signs^a^ | No | 69.2 (83) | 31.4 (37) | Ref | 0.188 |
|  | Yes | 52.9 (9) | 42.1 (8) | 2.0 (0.7-5.6) |  |
| Received antimicrobials from feed and chick traders | No | 37.2 (32) | 62.8 (54) | Ref | 0.160 |
|  | Yes | 25.5 (13) | 74.5 (38) | 0.6 (0.3-1.2) |  |
| Purchased antimicrobials from veterinary medical stores | No | 33.3 (16) | 66.7 (32) | Ref | 0.929 |
|  | Yes | 32.6 (29) | 67.4 (60) | 1.0 (0.5-2.0) |  |
| Purpose of using antimicrobial | Therapeutic | 14.3 (3) | 85.7 (18) | Ref | 0.002 |
|  | Prophylactic | 12.9 (4) | 87.1 (27) | 0.9 (0.2-4.5) |  |
|  | Both therapeutic and prophylactic | 44.7 (38) | 55.3 (47) | 4.9 (1.3-17.7) |  |
| Sale of antimicrobials by commercial chicken farmers | No | 33.0 (38) | 67.0 (77) | Ref | 0.911 |
|  | Yes | 31.8 (7) | 68.2 (15) | 0.9 (0.4-2.5) |  |

^a^This includes swollen head, ascites, in-appetence, and eye problems

**Table S6**. Results of the univariate analysis for risk factors associated with the use of doxycycline on commercial poultry farms in Bangladesh in 2019.

| **Risk factors** | **Category** | **Use of Doxycycline** | | **OR**  **(95% CI)** | **Logistic regression p-value** |
| --- | --- | --- | --- | --- | --- |
|  |  | **Yes** | **No** |  |  |
|  |  | **% (N)** | **% (N)** |  |  |
| Farm type | Layer | 25.9 (14) | 74.1 (40) | Ref | 0.005 |
|  | Broiler | 50.6 (42) | 49.4 (41) | 2.9 (1.4-6.2) |  |
| Flock size | ≤500 | 20.0 (1) | 80.0 (4) | Ref | 0.050 |
|  | 501-2500 | 47.1 (48) | 52.9 (54) | 3.6 (0.4-32.9) |  |
|  | >2500 | 23.3 (7) | 76.7 (23) | 1.2 (0.1-12.8) |  |
| Education | No education/  primary | 45.5 (10) | 54.5 (12) | Ref | 0.634 |
|  | Secondary/graduate/  post graduate | 40.0 (46) | 60.0 (69) | 0.8 (0.3-2.0) |  |
| Experience | 6 months -5 years | 51.4 (19) | 48.7 (18) | Ref | 0.131 |
|  | >5 years | 37.0 (37) | 63.0 (63) | 0.6 (0.3-1.2) |  |
| Presence of any clinical signs | No | 32.4 (11) | 67.7 (23) | Ref | 0.246 |
|  | Yes | 43.7 (45) | 56.3 (58) | 1.6 (0.7-3.7) |  |
| Occurrence of respiratory signs | No | 42.9 (27) | 57.1 (36) | Ref | 0.663 |
|  | Yes | 39.2 (29) | 60.8 (45) | 0.9 (0.4-1.7) |  |
| Occurrence of enteric signs | No | 40.4 (42) | 59.6 (62) | Ref | 0.836 |
|  | Yes | 42.4 (14) | 57.6 (19) | 1.1 (0.5-2.4) |  |
| Increased mortality | No | 42.5 (51) | 57.5 (69) | Ref | 0.309 |
|  | Yes | 29.4 (5) | 70.6 (12) | 0.6 (0.2-1.7) |  |
| Decreased egg production or poor quality | No | 59.5 (75) | 40.5 (51) | Ref | 0.748 |
|  | Yes | 54.6 (6) | 45.5 (5) | 1.2 (0.4-4.2) |  |
| Occurrence of single miscellaneous signs^a^ | No | 41.7 (50) | 58.3 (70) | Ref | 0.618 |
|  | Yes | 35.3 (6) | 64.7 (11) | 0.7 (0.3-2.2) |  |
| Received antimicrobials from feed and chick traders | No | 37.2 (32) | 62.8 (54) | Ref | 0.258 |
|  | Yes | 47.1 (24) | 52.9 (27) | 1.5 (0.7-3.0) |  |
| Purchased antimicrobials from veterinary medical stores | No | 48.3 (43) | 51.7 (46) | Ref | 0.017 |
|  | Yes | 27.1 (13) | 72.9 (35) | 0.4 (0.2-0.8) |  |
| Purpose of using antimicrobial | Therapeutic | 33.3 (7) | 66.8 (14) | Ref | 0.167 |
|  | Prophylactic | 29.0 (9) | 71.0 (22) | 0.8 (0.2-2.7) |  |
|  | Both therapeutic and prophylactic | 47.1 (40) | 52.9 (45) | 1.8 (0.7-4.8) |  |
| Sale of antimicrobials by commercial chicken farmers | No | 38.3 (44) | 61.7 (71) | Ref | 0.159 |
|  | Yes | 54.5 (12) | 45.5 (10) | 1.9 (0.8-4.9) |  |

^a^This includes swollen head, ascites, in-appetence, and eye problems

**Table S7.** Results of the univariate analysis for risk factors associated with the use of ciprofloxacin on commercial poultry farms in Bangladesh in 2019.

| **Risk factors** | **Category** | **Use of Ciprofloxacin** | | **OR**  **(95% CI)** | **Logistic regression p-value** |
| --- | --- | --- | --- | --- | --- |
|  |  | **Yes** | **No** |  |  |
|  |  | **% (N)** | **% (N)** |  |  |
| Farm type | Layer | 37.0 (20) | 63.0 (34) | Ref | 0.692 |
|  | Broiler | 33.7 (28) | 66.3 (55) | 0.9 (0.4-1.8) |  |
| Flock size | ≤500 | 40.0 (2) | 60.0 (3) | Ref | 0.955 |
|  | 501-2500 | 35.3 (36) | 64.7 (66) | 0.8(0.1-5.1) |  |
|  | >2500 | 33.3 (10) | 66.7 (20) | 0.8(0.1-5.2) |  |
| Education | No education/  primary | 31.8 (7) | 68.2 (15) | Ref | 0.730 |
|  | Secondary/graduate/  post graduate | 35.7 (41) | 64.4 (74) | 1.2 (0.4-3.1) |  |
| Experience | 6 months -5 years | 73.0 (27) | 27.0 (10) | Ref | 0.234 |
|  | >5 years | 62.0 (62) | 38.0 (38) | 1.7 (0.7-3.8) |  |
| Presence of any clinical signs reported | No | 23.5 (8) | 76.5 (26) | Ref | 0.109 |
|  | Yes | 38.8 (40) | 61.2 (63) | 2.1 (0.9-5.0) |  |
| Occurrence of respiratory signs | No | 27.0 (17) | 73.0 (46) | Ref | 0.070 |
|  | Yes | 41.9 (31) | 58.1 (43) | 2.0 (0.9-4.0) |  |
| Occurrence of enteric signs | No | 31.7 (33) | 68.3 (71) | Ref | 0.153 |
|  | Yes | 45.5 (15) | 54.5 (18) | 1.8 (0.8-4.0) |  |
| Increased mortality | No | 35.0 (42) | 65.0 (78) | Ref | 0.981 |
|  | Yes | 35.3 (6) | 64.7 (11)  ) | 1.0 (0.3-2.9) |  |
| Decreased egg production or poor quality | No | 64.3 (81) | 35.7 (45) | Ref | 0.576 |
|  | Yes | 72.7 (8) | 27.3 (3) | 0.7 (0.2-2.7) |  |
| Occurrence of single miscellaneous signs^a^ | No | 67.5 (81) | 32.5 (39) | Ref | 0.105 |
|  | Yes | 47.1 (8) | 52.9 (9) | 2.3 (0.8-6.5) |  |
| Received antimicrobials from feed and chick traders | No | 39.5 (34) | 60.5 (52) | Ref | 0.154 |
|  | Yes | 27.5 (14) | 72.5 (37) | 0.6 (0.3-1.2) |  |
| Purchased antimicrobials from veterinary medical stores | No | 33.7 (30) | 66.3 (59) | Ref | 0.657 |
|  | Yes | 37.5 (18) | 62.5 (30) | 1.2 (0.6-2.5) |  |
| Purpose of using antimicrobial | Therapeutic | 28.6 (6) | 71.4 (15) | Ref | 0.009 |
|  | Prophylactic | 12.9 (4) | 87.1 (27) | 0.3 (0.1-1.5) |  |
|  | Both therapeutic and prophylactic | 44.7 (38) | 55.3 (47) | 2.0 (0.7-5.7) |  |
| Sale of antimicrobials by commercial chicken farmers | No | 36.5 (42) | 63.5 (73) | Ref | 0.393 |
|  | Yes | 27.3 (6) | 72.7 (16) | 1.2 (0.8-1.7) |  |

^a^This includes swollen head, ascites, in-appetence, and eye problems

**Table S8.** Results of the univariate analysis for risk factors associated with the use of tylosin on commercial poultry farms in Bangladesh in 2019.

| **Risk factors** | **Category** | **Use of Tylosin** | | **OR**  **(95% CI)** | **Logistic regression p-value** |
| --- | --- | --- | --- | --- | --- |
|  |  | **Yes** | **No** |  |  |
|  |  | **% (N)** | **% (N)** |  |  |
| Farm type | Layer | 16.7 (9) | 83.3 (45) | Ref | 0.578 |
|  | Broiler | 20.5 (17) | 79.5 (66) | 1.2 (0.5-3.1) |  |
| Flock size | ≤500 | 0.0 (0) | 100.0 (5) | - | - |
|  | 501-2500 | 22.6 (23) | 77.5 (79) | - |  |
|  | >2500 | 10.0 (3) | 90.0 (27) | - |  |
| Education | No education/  primary | 17.4 (20) | 82.6 (95) | Ref | 0.283 |
|  | Secondary/graduate/  post graduate | 27.3 (6) | 72.7 (16) | 0.6 (0.2-1.6) |  |
| Experience | 6 months -5 years | 10.8 (4) | 89.2 (33) | Ref | 0.147 |
|  | >5 years | 22.0 (22) | 78.0 (78) | 2.3 (0.7-7.3) |  |
| Presence of any clinical signs reported | No | 8.8 (3) | 91.2 (31) | Ref | 0.094 |
|  | Yes | 22.3 (23) | 77.7 (80) | 3.0 (0.8-10.6) |  |
| Occurrence of respiratory signs | No | 17.5 (11) | 82.5 (52) | Ref | 0.676 |
|  | Yes | 20.3 (15) | 79.7 (59) | 1.2 (0.5-2.8) |  |
| Occurrence of enteric signs | No | 16.4 (17) | 83.7 (87) | Ref | 0.168 |
|  | Yes | 27.3 (9) | 72.7 (24) | 1.9 (0.8-4.8) |  |
| Increased mortality | No | 20.0 (24) | 80.0 (96) | Ref | 0.424 |
|  | Yes | 11.8 (2) | 88.2 (15) | 0.5 (0.1-2.5) |  |
| Decreased egg production or poor quality | No | 18.3 (23) | 81.8 (103) | Ref | 0.469 |
|  | Yes | 27.3 (3) | 72.7 (8) | 1.7 (0.4-6.8) |  |
| Occurrence of single miscellaneous signs^a^ | No | 16.7 (20) | 83.3 (100) | Ref | 0.075 |
|  | Yes | 35.3 (6) | 64.7 (11) | 2.7 (0.9-8.2) |  |
| Received antimicrobials from feed and chick traders | No | 17.4 (15) | 82.6 (71) | Ref | 0.552 |
|  | Yes | 21.6 (11) | 78.4 (40) | 1.3 (0.5-3.1) |  |
| Purchased antimicrobials from veterinary medical stores | No | 19.1 (17) | 80.9 (72) | Ref | 0.960 |
|  | Yes | 18.8 (9) | 81.2 (39) | 0.9 (0.4-2.4) |  |
| Purpose of using antimicrobial | Therapeutic | 28.6 (6) | 71.4 (15) | Ref | 0.129 |
|  | Prophylactic | 6.5 (2) | 93.5 (29) | 0.2 (0.1-0.9) |  |
|  | Both therapeutic and prophylactic | 21.2 (18) | 78.8 (67) | 0.7 (0.2-2.0) |  |
| Sale of antimicrobials by commercial chicken farmers | No | 20.9 (24) | 79.1 (91) | Ref | 0.212 |
|  | Yes | 9.1 (2) | 90.9 (20) | 0.4 (0.1-1.7) |  |

^a^This includes swollen head, ascites, in-appetence, and eye problems

**Table S9**. Results of the univariate analysis for risk factors associated with the use of trimethoprim sulphonamides on commercial poultry farms in Bangladesh in 2019.

| **Risk factors** | **Category** | **Use of Trimethoprim sulphonamides** | | **OR**  **(95% CI)** | **Logistic regression p-value** |
| --- | --- | --- | --- | --- | --- |
|  |  | **Yes** | **No** |  |  |
|  |  | **% (N)** | **% (N)** |  |  |
| Farm type | Layer | 29.6 (16) | 70.4 (38) | Ref | 0.117 |
|  | Broiler | 18.1 (15) | 81.9 (68) | 0.5 (0.2-1.2) |  |
| Flock size | ≤500 | 20.0 (1) | 80.0 (4) | Ref | 0.985 |
|  | 501-2500 | 22.6 (23) | 77.5 (79) | 1.2 (0.1-10.9) |  |
|  | >2500 | 23.3 (7) | 76.7 (23) | 1.2 (0.1-12.8) |  |
| Education | No education/  primary | 36.4 (8) | 63.6 (14) | Ref | 0.099 |
|  | Secondary/graduate/  post graduate | 20.0 (23) | 80.0 (92) | 0.4 (0.2-1.2) |  |
| Experience | 6 months -5 years | 18.9 (7) | 81.1 (30) | Ref | 0.529 |
|  | >5 years | 24.0 (24) | 76.0 (76) | 1.4 (0.5-3.5) |  |
| Presence of any clinical signs reported | No | 11.8 (4) | 88.2 (30) | Ref | 0.090 |
|  | Yes | 26.2 (27) | 73.8 (76) | 2.7 (0.9-8.2) |  |
| Occurrence of respiratory signs | No | 12.7 (8) | 87.3 (55) | Ref | 0.013 |
|  | Yes | 31.1 (23) | 68.9 (51) | 3.1 (1.3-7.6) |  |
| Occurrence of enteric signs | No | 17.3 (18) | 82.7 (86) | Ref | 0.010 |
|  | Yes | 39.4 (13) | 60.6 (20) | 3.1 (1.3-7.4) ) |  |
| Increased mortality | No | 23.3 (28) | 76.7 (92) | Ref | 0.601 |
|  | Yes | 17.6 (3) | 82.4 (14) | 0.7 (0.2-2.6) |  |
| Decreased egg production or poor quality | No | 23.0 (29) | 77.0 (97) | Ref | 0.714 |
|  | Yes | 18.2 (2) | 81.8 (9 | 0.7 (0.2-3.6) |  |
| Occurrence of single miscellaneous signs^a^ | No | 23.3 (28) | 76.7 (92) | Ref | 0.601 |
|  | Yes | 17.6 (3) | 82.4 (14) | 0.7 (0.2-2.6) |  |
| Received antimicrobials from feed and chick traders | No | 25.6 (22) | 79.8 (71) | Ref | 0.286 |
|  | Yes | 17.7 (9) | 72.9 (35) | 0.6 (0.3-1.5) |  |
| Purchased antimicrobials from veterinary medical stores | No | 20.2 (18) | 79.8 (71) | Ref | 0.361 |
|  | Yes | 27.1 (13) | 72.9 (35) | 1.5 (0.6-3.3) |  |
| Purpose of using antimicrobial | Therapeutic | 42.9 (9) | 57.1 (12) | Ref | 0.067 |
|  | Prophylactic | 19.4 (6) | 80.7 (25) | 0.3 (0.1-1.1) |  |
|  | Both therapeutic and prophylactic | 18.8 (16) | 81.2 (69) | 0.3 (0.1-0.9) |  |
| Sale of antimicrobials by commercial chicken farmers | No | 22.6 (26) | 77.4 (89) | Ref | 0.990 |
|  | Yes | 22.7 (5) | 77.3 (17) | 1.0 (0.3-3.0) |  |

^a^This includes swollen head, ascites, in-appetence, and eye problems

**Table S10.** Results of the univariate analysis for risk factors associated with the use of neomycin on commercial poultry farms in Bangladesh in 2019.

| **Risk factors** | **Category** | **Use of Neomycin** | | **OR**  **(95% CI)** | **Logistic regression p-value** |
| --- | --- | --- | --- | --- | --- |
|  |  | **Yes** | **No** |  |  |
|  |  | **% (N)** | **% (N)** |  |  |
| Farm type | Layer | 7.4 (4) | 92.6 (50) | Ref | 0.000 |
|  | Broiler | 38.6 (32) | 61.5 (51) | 7.8 (2.6-23.8) |  |
| Flock size | ≤500 | 0.0 (0) | 100.0 (5) | - | - |
|  | 501-2500 | 33.3 (34) | 66.7 (68) | - |  |
|  | >2500 | 6.7 (2) | 93.3 (28) | - |  |
| Education | No education/  primary | 45.5 (10) | 54.5 (12) | Ref | 0.030 |
|  | Secondary/graduate/  post graduate | 22.6 (26) | 77.4 (89) | 0.4 (0.1-0.9) |  |
| Experience | 6 months -5 years | 40.5 (15) | 59.5 (22) | Ref | 0.023 |
|  | >5 years | 21.0 (21) | 79.0 (79) | 0.4 (0.2-0.9) |  |
| Presence of any clinical signs reported | No | 26.5 (9) | 73.5 (25) | Ref | 0.976 |
|  | Yes | 26.2 (27) | 73.8 (76) | 1.0 (0.4-2.3) |  |
| Occurrence of respiratory signs | No | 30.2 (19) | 69.8 (44) | Ref | 0.342 |
|  | Yes | 23.0 (17) | 77.0 (57) | 0.7 (0.3-1.5) |  |
| Occurrence of enteric signs | No | 27.9 (29) | 72.1 (75) | Ref | 0.450 |
|  | Yes | 21.2 (7) | 78.8 (26) | 0.7 (0.3-1.8) |  |
| Increased mortality | No | 26.7 (32) | 73.3 (88) | Ref | 0.783 |
|  | Yes | 23.5 (4) | 76.5 (13) | 0.8 (0.3-2.8) |  |
| Decreased egg production or poor quality | No | 27.0 (34) | 73.0 (92) | Ref | 0.529 |
|  | Yes | 18.2 (2) | 81.8 (9) | 0.6 (0.1-2.9) |  |
| Occurrence of single miscellaneous signs^a^ | No | 26.7 (32) | 73.3 (88) | Ref | 0.783 |
|  | Yes | 23.5 (4) | 76.5 (13) | 0.8 (0.3-2.8) |  |
| Received antimicrobials from feed and chick traders | No | 26.7 (23) | 73.3 (63) | Ref | 0.872 |
|  | Yes | 25.5 (13) | 79.2 (38) | 0.9 (0.4-2.1) |  |
| Purchased antimicrobials from veterinary medical stores | No | 29.2 (26) | 70.8 (63) | Ref | 0.290 |
|  | Yes | 20.8 (10) | 79.2 (38) | 0.6 (0.3-1.5) |  |
| Purpose of using antimicrobial | Therapeutic | 23.8 (5) | 76.2 (16) | Ref | 0.953 |
|  | Prophylactic | 25.8 (8) | 74.2 (23) | 1.1 (0.3-4.0) |  |
|  | Both therapeutic and prophylactic | 27.1 (23) | 72.9 (62) | 1.2 (0.4-3.6) |  |
| Sale of antimicrobials by commercial chicken farmers | No | 22.6 (26) | 77.4 (89) | Ref | 0.030 |
|  | Yes | 45.5 (10) | 54.5 (12) | 2.9 (1.1-7.3) |  |

^a^This includes swollen head, ascites, in-appetence, and eye problems

**Table S11.** Results of the univariate analysis for risk factors associated with the use of colistin on commercial poultry farms in Bangladesh in 2019.

| **Risk factors** | **Category** | **Use of Colistin** | | **OR**  **(95% CI)** | **Logistic regression p-value** |
| --- | --- | --- | --- | --- | --- |
|  |  | **Yes** | **No** |  |  |
|  |  | **% (N)** | **% (N)** |  |  |
| Farm type | Layer | 27.8 (15) | 72.2 (39) | Ref | 0.001 |
|  | Broiler | 56.6 (47) | 43.4 (36) | 3.4 (1.6-7.1) |  |
| Flock size | ≤500 | 40.0 (2) | 60.0 (3) | Ref | 0.027 |
|  | 501-2500 | 52.0 (53) | 48.0 (49) | 1.6 (0.5-5.2) |  |
|  | >2500 | 23.3 (7) | 76.7 (23) | 0.5 (0.1-3.3) |  |
| Education | No education/  primary | 54.5 (12) | 45.5 (10) | Ref | 0.342 |
|  | Secondary/graduate/  post graduate | 43.5 (50) | 56.5 (65) | 0.6 (0.3-1.6) |  |
| Experience | 6 months -5 years | 51.4 (19) | 48.7 (18) | Ref | 0.384 |
|  | >5 years | 43.0 (43) | 57.0 (57) | 0.7 (0.3-1.5) |  |
| Presence of any clinical signs reported | No | 47.1 (16) | 52.9 (18) | Ref | 0.808 |
|  | Yes | 44.7 (46) | 55.3 (57) | 0.9 (0.4-2.0) |  |
| Occurrence of respiratory signs | No | 49.2 (31) | 50.8 (32) | Ref | 0.392 |
|  | Yes | 41.9 (31) | 58.1 (43) | 0.7 (0.4-1.5) |  |
| Occurrence of enteric signs | No | 44.2 (46) | 55.8 (58) | Ref | 0.669 |
|  | Yes | 48.5 (16) | 51.5 (17) | 1.2 (0.5-2.6) |  |
| Increased mortality | No | 47.5 (57) | 52.5 (63) | Ref | 0.168 |
|  | Yes | 29.4 (5) | 70.6 (12) | 0.5 (0.2-1.4) |  |
| Decreased egg production or poor quality | No | 46.0 (58) | 54.0 (68) | Ref | 0.539 |
|  | Yes | 36.4 (4) | 63.6 (7) | 0.7 (0.2-2.4) |  |
| Occurrence of single miscellaneous signs^a^ | No | 42.5 (51) | 57.5 (69) | Ref | 0.093 |
|  | Yes | 64.7 (11) | 35.3 (6) | 2.5 (0.9-7.1) |  |
| Received antimicrobials from feed and chick traders | No | 40.7 (35) | 59.3 (51) | Ref | 0.165 |
|  | Yes | 52.9 (27) | 69.2 (18) | 1.6 (0.8-3.3) |  |
| Purchased antimicrobials from veterinary medical stores | No | 40.7 (35) | 48.3 (43) | Ref | 0.041 |
|  | Yes | 52.9 (27) | 66.7 (32) | 0.5 (0.2-1.0) |  |
| Purpose of using antimicrobial | Therapeutic | 28.6 (6) | 71.4 (15) | Ref | 0.121 |
|  | Prophylactic | 38.7 (12) | 61.3 (19) | 1.5 (0.5-5.2) |  |
|  | Both therapeutic and prophylactic | 51.8 (44) | 48.2 (41) | 2.7 (0.9-7.6) |  |
| Sale of antimicrobials by commercial chicken farmers | No | 45.2 (52) | 54.8 (63) | Ref | 0.984 |
|  | Yes | 45.5 (10) | 54.5 (12) | 1.0 (0.4-2.5) |  |

^a^This includes swollen head, ascites, in-appetence, and eye problems

**Table S12.** Results of the univariate analysis for risk factors associated with the use of tiamulin on commercial poultry farms in Bangladesh in 2019.

| **Risk factors** | **Category** | **Use of Tiamulin** | | **OR**  **(95% CI)** | **Logistic regression p-value** |
| --- | --- | --- | --- | --- | --- |
|  |  | **Yes** | **No** |  |  |
|  |  | **% (N)** | **% (N)** |  |  |
| Farm type | Layer | 31.5 (17) | 68.5 (37) | - | - |
|  | Broiler | 0.0 (0) | 100.0 (83) | - |  |
| Flock size | ≤500 | 0.0 (0) | 100.0 (5) | - | - |
|  | 501-2500 | 7.8 (8) | 92.2 (94) | - |  |
|  | >2500 | 30.0 (9) | 70.0 (21) | - |  |
| Education | No education/  primary | 9.1 (2) | 90.9 (20) | Ref | 0.609 |
|  | Secondary/graduate/  post graduate | 13.0 (15) | 87.0 (100) | 1.5 (3.0-7.1) |  |
| Experience | 6 months -5 years | 5.4 (2) | 94.6 (35) | Ref | 0.148 |
|  | >5 years | 15.0 (15) | 85.0 (85) | 3.1 (0.7-14.2) |  |
| Presence of any clinical signs reported | No | 11.8 (4) | 88.2 (30) | Ref | 0.896 |
|  | Yes | 12.6 (13) | 87.4 (90) | 1.1 (0.3-3.6) |  |
| Occurrence of respiratory signs | No | 11.1 (7) | 88.9 (56) | Ref | 0.671 |
|  | Yes | 13.5 (10) | 86.5 (64) | 1.3 (0.5-3.5) |  |
| Occurrence of enteric signs | No | 13.5 (14) | 86.5 (90) | Ref | 0.510 |
|  | Yes | 9.1 (3) | 90.9 (30) | 0.6 (0.2-2.4) |  |
| Increased mortality | No | 13.3 (16) | 86.7 (104) | Ref | 0.398 |
|  | Yes | 5.9 (1) | 94.1 (16) | 0.4 (0.1-3.3) |  |
| Decreased egg production or poor quality | No | 9.5 (12) | 90.5 (114) | Ref | 0.002 |
|  | Yes | 45.5 (5) | 54.6 (6) | 7.9 (2.1-29.9) |  |
| Occurrence of single miscellaneous signs^a^ | No | 14.2 (17) | 85.8 (103) | - | - |
|  | Yes | 0.0 (0) | 100.0 (17) | - |  |
| Received antimicrobials from feed and chick traders | No | 15.1 (13) | 84.9 (73) | Ref | 0.220 |
|  | Yes | 7.8 (4) | 92.2 (47) | 0.5 (0.1-1.6) |  |
| Purchased antimicrobials from veterinary medical stores | No | 11.2 (10) | 88.8 (79) | Ref | 0.572 |
|  | Yes | 14.6 (7) | 85.4 (41) | 1.3 (0.5-3.8) |  |
| Purpose of using antimicrobial | Therapeutic | 9.5 (2) | 90.5 (19) | Ref | 0.419 |
|  | Prophylactic | 19.4 (6) | 80.7 (25) | 2.3 (0.4-12.6) |  |
|  | Both therapeutic and prophylactic | 10.6 (9) | 89.4 (76) | 1.1 (0.2-5.6) |  |
| Sale of antimicrobials by commercial chicken farmers | No | 11.3 (13) | 88.7 (102) | Ref | 0.375 |
|  | Yes | 18.2 (4) | 81.8 (18) | 1.7 (0.5-6.0) |  |

^a^This includes swollen head, ascites, in-appetence, and eye problems
