# Supplementary material for: A Cross-Sectional Study of Antimicrobial Usage on Commercial Broiler and Layer Chicken Farms in Bangladesh
Source: Front Vet Sci. 2020 Dec 16;7:576113. doi: 10.3389/fvets.2020.576113 (PMC7772320; doi:10.3389/fvets.2020.576113)
Supplement: Supplementary file 2 [file Data_Sheet_1.pdf]

Assessment of antimicrobial usage on commercial poultry farms and, attitudes and behaviours of antimicrobial usage by commercial poultry farmers and attitudes and behaviours of antimicrobial sales and distribution by traders of antimicrobials in Bangladesh

Demographic/Socioeconomic characteristics of the interviewee

(Tick the boxes and fill in the blanks)

**1. Date of interview:**	___________ (day)	__________(month)	__________(Year)
**2. Farm ID**			
**3. Name of the interviewee:**			
**4. What is your farm type?**	0= Meat type (Broiler) 1= Egg type (Layer)		
**5. Status of the interviewee on farm:**	0=Owner<br>1=Manager<br>2=Worker<br>3=Owner's spouse<br>4=Owner's son<br>5=Owner's daughter<br>6=Other __________________		

Poultry Information

**6. How many chickens do you have in the farm today?**			
**7. What is your current production system?**	0=All-in-All out	1=Continuous	2=Both
**8. How many sheds you have in your farm?**			
**9. Do you use antimicrobials/antibiotics/medicines/ vitamins/minerals in your farm?**	0=No	1=Yes	
**9.1. If yes, do you use different amount of antimicrobials/medicines/vitamins/antibiotics in different sheds?**	0=No	1=Yes	
**9.1.1. If yes, in which shed is the highest amount of antimicrobial/medicine/vitamins or antibiotics used?**<br><br>**THIS IS THE SHED TO BE SAMPLED (if we get ans here then ques 12 will not appear)**	0=Shed 1<br>3= Shed 4	1= Shed 2<br>4= Shed 5	2=Shed 3<br>5=Other shed (specify)____
**9.1.2. If no,do you have birds of different age on your farm?**	0=No	1=Yes	
**9.1.2.1. If yes, in which shedare the oldest birds? THIS IS THE SHED TO BE SAMPLED**<br><br>**If no (all birds are of the same age) then THE SHED TO BE SAMPLED will be selected randomly.**	0=Shed 1<br>3= Shed 4	1= Shed 2<br>4= Shed 5	2=Shed 3<br>5=Shed 6<br>6=Other shed (specify)____
**10. How many chickens you have in the shed today from which faecal sample is taken (from sampled shed faecal sample will be collected)?**			

**11. What is the age of the poultry in the shed from which faecal sample is collected?**	_________(day)	______(month)	______ (Year)
**12. What are the ages of the poultry from other sheds?**			
**12.1. If, all in all out, then collect the age for one batch (as all the chickens are of same age, so all sheds will be of same ages)**			
**12.2. If, continuous then collect age for different batches**			
**12.2.1. 1st Shed of same age**	__________ (day)	________ (month)	_________ (Year)
**12.2.2. 2nd Shed of same age**	__________ (day)	________ (month)	_________ (Year)
**12.2.3. 3rd Shed of same age**	__________ (day)	________ (month)	_________ (Year)
**12.2.4. 4th Shed of same age**	__________ (day)	________ (month)	_________ (Year)
**12.2.5. 5th Shed of same age**	__________ (day)	________ (month)	_________ (Year)
**12.2.6. 6th Shed of same age**	__________ (day)	________ (month)	_________ (Year)
**12.2.7. Others____________**			

Antimicrobial/medicine/vitamins data

Sampled Shed

Batch: Current batch

**13. Can you show the drugs/medicines/antimicrobials/ antibiotics/minerals/vitamins you used in the sampled shed in the current production cycle?**			
**14. What is the active ingredient of the drug? (will be answered by the interviewer after they have a look at the drug)**			
**15. Picture of the drugs used (if any)**			
**16. Why/when you use drugs/antimicrobials /medicines/ vitamins/antibiotics/ for your poultry?**			
**17. What is the purpose of using antimicrobials/medicinse/vitamins/ drugs in current batch of the poultry? (select the shed from which faecal sample is collected)**	0=Therapeutic<br>3=Both therapeutic and preventive	1=Prophylactic<br>4=Others (specify)________	2=Growth promotion
**18. How many times you use antimicrobial/medicine/vitamin in that sampled shed?**	0=Once daily	1=Twice daily	2=thrice
**19. Is the dose same at each time? (this ques is linked to ques 18)**	0=No	1=Yes	
**20. What is the actual dose rate at each administration (take the dose for each**			

antimicrobial used)?			
**21. What is the duration of usage (in days) in the sampled shed (take the dose for each antimicrobial used)?**			
**22. What is the route of administration?**	0=Feed	1=Water	2=Both feed and water
**23. What clinical signs did you observe in this sampledflock in which you used the antimicrobial? (for therapeutic)**	0=coughing	1= breathing difficulty	2= sneezing
	3= faeces with blood	4= green faeces	5= feather problems
	6= eye problems	7= nasal problems	8= poor growth
	9=less egg production (for layer)	10= increase in early mortality	11=depression/in apetance
	12= loose droppings	13= chalky faeces	14= egg deformation
	15=swollen head	16= Others (specify)______	
**24. What purpose are the antimicrobials used preventively – to prevent what? (for prophylactic)**			
**25. For whatdiseases suspected in this sampledflock did you use the antimicrobial?**	0= Salmonellosis<br>3= Avian Influenza<br>6= Avian leucosis<br>9= Mycoplasmosis<br>12 = Necrotic enteritis	1= Colibacillosis<br>4= Newcastle<br>7= Infectious bursal disease<br>10 = Egg peritonitis<br>13 = Ingectious coryza	2= Fowl cholera<br>5= Coccidiosis<br>8 =Infectious bronchitis (IB)<br>11 = Helminth parasitic infection<br>14=Others (specify)________
**26. In case of broiler,do you stop using the antimicrobial/medicine/vitamin before sale of birds**	0= No 1= Yes		
**26.1. If yes, how many days before the sale do you stop using antimicrobial/medicine/vitamins (for what specific product)?**			
**26.2. For layers, do you sell eggs while antimicrobia/medicine/drugs/minerals/vitamins/antibiotics is being used?**	0= No 1= Yes		
**27. Who advised to treat the affected poultry?**	0=Government Veterinary doctor	1=Private Veterinary doctor	2= Feed chick dealer
	3=Medical representatives	4=Others (specify) ______	
**28. Who provided you with the antimicrobial/medicine/vitamins?**	0=Pharmacy	1=Feed and chick dealer	2=Government Veterinarian
	3=Private Veterinarian	4=Medical representatives	5=Others ___
**28.1. Provider's name**			
**28.2. Provider's contact number**			
**29. When the poultry are found sick with disease what do you do?**			

Shed: Sampled shed(past information)

**30. Have you used any other products in the sampled shed in last batch?**	0=No 1=Yes		
**If no,then no ques will be asked, if yes then ques 32-39 will be asked**			
**31. Why did you use AM?**	0=Therapeutic<br><br>3=Both therapeutic and preventive	1=Preventive/ Prophylactic<br><br>4=Others(specify) _____	2=Growth promoting
**32. What was the name of the product?**			
**33. Active ingredient (answered by the interviewer)**			
**34. How many times you use antimicrobial/medicine/vitamins in that sampled shed?**	0=Once daily	1=Twice daily	2=thrice
**35. Is the dose same at each time? (this ques is linked to ques 35)**	0=No	1=Yes	
**35.1. If no, then what is the amount you administer each time? (If AM is given once then this ques will not appear, if AM is given more than once it will appear)**	If in ques 35, it is clicked once then 1st time dose________	If in ques 35, it is clicked twice then 1st time dose________<br><br>2nd time dose________ Timing _____ hours	If in ques 35, it is clicked hrice then 1st time dose________<br><br>2nd time dose_______ Timing _____ hours<br><br>3rd time dose________ Timing _____ hours
**36. What is the route of administration?**	0=Feed<br><br>3=Injection	1=Water<br><br>4=Others ______	2=Both feed and water
**37. In case of broiler,did you stop using the antimicrobial/vitamins/drugs/medicine/ antibiotic before sale of birds ?**	0=No 1=Yes		
**37.1. If yes, then how many days after the antimicrobial/medicine/vitamins administration date?**			
**38. For layers, did you sell eggs while antimicrobial/vitamins/drugs/medicine/ antibiotic is being used?**	0= No	1= Yes	

**Other sheds**			
**39. Do you use currently any other products in other sheds?**	0= No	1= Yes	
**If no, then no questions will be asked, if yes, then 40-48 will be asked**			
**40. Why do you use AM?**	0=Therapeutic	1= Prophylactic	2=Growth promoting<br><br>3=Both therapeutic and preventive
**41. Can you show me the product you are using in other sheds?**			
**42. Active ingredient (answered by the interviewer)**			
**43. How many times you use antimicrobial/medicine/vitamins in that sampled shed?**	0=Once daily	1=Twice daily	2=thrice
**44. Is the dose same at each time?**	0=No	1=Yes	
**45. If no, then what is the amount you administer each time? (If AM is given once then this ques will not appear, if AM is given more than once it will appear)**	If in ques 23, it is clicked once then 1st time dose________	If in ques 23, it is clicked twice then 1st timedose____<br><br>2nd timedose____ Timing ___ hours	If in ques 23, it is clicked hrice then 1st time dose________<br><br>2nd time dose_______ Timing ___ hours<br><br>3rd time dose___ Timing ___ hours
**46. What is the route of administration?**	0=Feed<br>3=Injection	1=Water<br>4=Others ________	2=Both feed and water
**47. In case of broiler,do you stop using the antimicrobial/medicine/vitamins before sale of birds**	0=No	1=Yes	
**47.1. If yes, then how many days after the antimicrobial/medicine/vitamins administration date?**			
**48. For layers, do you sell eggs while antimicrobial/medicine/vitamins is being used**	0=No	1=Yes	

Attitude and behaviour of farmers towards antimicrobial usage

#	Statement	Strongly agree	Agree	Strongly disagree	Disagree	Don't know
	**Knowledge about antimicrobials**					
**1.**	Giving antimicrobials to healthy chickens makes them less likely to become sick					
**2.**	Antimicrobials use in chickens is necessary to control diseases in humans					
**3.**	Antimicrobials can be used in chickens any dosage, route and frequency					
**4.**	Antimicrobials and vaccines are the same products					
	**Risks of AM usage**					
**5.**	Antimicrobials residues in chicken meat will not harm humans					
**6.**	Antimicrobials provided to chickens will be destroyed during cooking and frying					
**7.**	Antimicrobials can be given to chickens up to the day before selling					
**8.**	If an antimicrobials has healed my chickens once, it will always be effective to heal the same disease in the future.					
**9.**	If antimicrobials is used in chicken, it will stay in chickens andwon't be excreted					
	**Behaviours regrding AM usage**					
**10.**	I am increasing thedosage of antimicrobials when I am experiencing more chicken getting sick or dying					
**11.**	I like to use multiple antimicrobials products to treat my chickens					
**12.**	I always have a range of antimicrobials availbale on my farm, even if don't used them all					
**13.**	I am increasing the dosage of antimicrobials when egg production decreases					
**14.**	I am increasing the dosage of antimicrobials when my broilers do not grow fast enough					
	**Perceived barriers for AM usage**					
**15.**	Antimicrobials are expensive to buy					
**16.**	There is a lack of clear national guidelines on how to use antimicrobials in chickens					
**17.**	It is difficult to find time to maintain good chickenhealth, so providing antimicrobials help me to save my valuable time					
**18.**	The labels of antimicrobial products do not provide clear information on how to use the antimicrobials					
	**Perceived benefits for AM usage**					
**19.**	Antimicrobials lead to a healthy growth of chickens					
**20.**	Antimicrobials help chickens to recover from disease					

**21.**	Antimicrobials help increase the egg production					
**22.**	Antimicrobial use in broilers will improve the quality of the chicken meat					
**23.**	Antimicrobial use in chickens will improve human health					
	**Self-Efficacy in AM usage**					
**24.**	I am skilled enough to select and administer antimicrobials without anyone's advice					
**25.**	I would invest time to participate in training on the proper use of antimicrobials					
**26.**	I believe that stronger laws and enforcement of the law are needed to reduce antimicrobials usage					
**27.**	I would invest time and money to further improve farm hygience and biosecurity to reduce the usage of antimicrobials on my farm					
**28.**	I am bound to take advice from feed chick dealers because I owe them money (they provide day old chicks, antimicrobials and feed)					
**29.**	I am not sure what to do when someone gives me advice about the usage of antimicrobials in chickens					

**Source of information on the usage of antimicrobials (dosage, frequency, types)**	Source not used	Only discussion, no advice received	Advice is not followed	Advice is rarely followed	Advice is frequently followed	Advice is always followed
Feed chick dealers						
Veterinarians						
Pharmaceutical representatives						
Members of the poultry association						
Human doctors						
Relatives (no poultry farmers)						
Relatives (poultry farmers)						
Neighbours (no poultry farmers)						
Neighbours (poultry farmers)						
Other commercial poultry farmers						
Others (specify)						

Biosecurity

(answers will be observed/asked by the interviewer)

**1.** Is the farm surrounded by a protective fence?	0=No	1=Yes	
**2.** In addition to the people involved in rearing poultry (listed in ques 5),who has access to your farm?	0=Feed suppliers	1=Other farm owners	2=Other farm workers
	3=Relatives	4=Feed chick dealer	5= Government Veterinarians
	6= Private Veterinarians	7= Poultry vaccinator	8= Owner/worker from another farm
	9= Others _______		
**3.** Does anyone who are involved in poultry keeping go to other commercial poultry farms?	0=No	1=Yes	
**3.1.** If yes in question 53, then how frequently does he/they visit in the last month?	0=daily	1=consequtive days	2=once in a week
	3=once in a fortnight	4=once in a month	5=others ______

**(Tick appropriate answers)**	**Yes**	**No**
**4.** Do you isolate/separate the sick birds in a separate shed?		
**5.** What do you do with dead birds?		
**6.** What do you do with your manure?		
**7.** Do the visitors/employees use washing facility before entering farm/shed?		
**8.** Do the employees change clothes and shoes before entering the farm/shed?		
**9.** Do the visitors change clothes and shoes before entering the farm/shed?		
**10.** Are the vehicles checked and decontaminated before entering farm?		
**11.** Are the vehicles decontaminated when leaving the farm?		
**12.** Do you have footbaths used, water changed and/or disinfectant within 6 hours?		
**13.** What types of water you allow for drinking, cleaning, washing or cooling at the farm?		
**14.** Do you weekly clean and/or disinfect the farm surfaces and equipments?		
**15.** Are egg trays washed when bringing back from market?		
**16.** Are farm employees given training on biosecurity measures?		
**17.** Do farm workers live within the farm premises?		
**17.1.** If yes, do they rear their own poultry birds within the farm premises?		

Other demographic and Farm informations

**1. Mobile number of the interviewee:**			
**Address of the farm:**			
a. Name of the poultry farm:			
b. Village:			
c. Ward:			
d. Union			
e. Upazilla/Thana:			
f. Latitude:			
g. Longitude:			
**2. Experience of the interviewee in poutry farming:**	0=< 6 months	1= (6-12) months<br>2= (1-5) years	3= (6-10) years<br>4=>10 years
**3. Age (in years)**			
**4. Gender:**	0=Male	1=Female	
**5. Education:**	0=No education	1=Up to Primary	2=Up to Secondary
	3=Up to higher secondary	4=Graduate	5=Post graduate
	6=Dakhil	7=Fazil	
**6. Marital status:**	0=Single	1=Married	2=Divorced
	3=Widow	4=Others ______	
**7. Religion:**	0=Muslim	1=Hindu	2=Christian
	3=Buddhist		
**8. Which is the source provides the largest income to your household ?**	0= Poultry rearing	1=Livestock rearing	2=Fishing
	3= Daily worker	4= Grocery	5= works at Non Government Organization
	6= Family business	7= Agriculture	8= Others_____
**9. Monthly Net Income (in BDT)**			
**10. What type of breed/strain you have in the farm currently? (THIS QUES will come if interviewer tick egg type)**	0=Novogen Brown<br>3= ISA Brown<br>6= Others_______	1= White Hyline Brown<br>4= Hi-Sex Brown	2= White Shaver 579<br>5=White Bovine White
**11. What type of breed/strain you have in the farm currently? (THIS QUES will come if interviewer tick meat type)**	1=Cobb 500<br>4= Hubbard classic	2=Ross 308<br>5=Arber acre	3= Indian River Meat<br>6= Others_____
